# Fouling Reduction and Thermal Efficiency Enhancement in Membrane Distillation Using a Bilayer-Fluorinated Alkyl Silane–Carbon Nanotube Membrane

**DOI:** 10.3390/membranes14070152

**Published:** 2024-07-10

**Authors:** Sumona Paul, Mitun Chandra Bhoumick, Somenath Mitra

**Affiliations:** Department of Chemistry and Environmental Science, New Jersey Institute of Technology, Newark, NJ 07102, USA; sp2652@njit.edu (S.P.); mb777@njit.edu (M.C.B.)

**Keywords:** FAS, carbon nanotubes, superhydrophobic, desalination, inorganic salt, fouling, membrane distillation

## Abstract

In this study, we report the robust hydrophobicity, lower fouling propensity, and high thermal efficiency of the 1H,1H,2H,2H-perfluorooctyltriethoxysilane (FAS)-coated, carbon nanotube-immobilized membrane (CNIM) when applied to desalination via membrane distillation. Referred to as FAS-CNIM, the membrane was developed through a process that combined the drop-casting of nanotubes flowed by a dip coating of the FAS layer. The membranes were tested for porosity, surface morphology, thermal stability, contact angle, and flux. The static contact angle of the FAS-CNIM was 153 ± 1°, and the modified membrane showed enhancement in water flux by 18% compared to the base PTFE membrane. The flux was tested at different operating conditions and the fouling behavior was investigated under extreme conditions using a CaCO_3_ as well as a mixture of CaCO_3_ and CaSO_4_ solution. The FAS-CNIM showed significantly lower fouling than plain PTFE or the CNIM; the relative flux reduction was 34.4% and 37.6% lower than the control for the CaCO_3_ and CaCO_3_/CaSO_4_ mixed salt solution. The FAS-CNIM exhibited a notable decrease in specific energy consumption (SEC). Specifically, the SEC for the FAS-CNIM measured 311 kwh/m^3^ compared to 330.5 kwh/m^3^ for the CNIM and 354 kwh/m^3^ for PTFE using a mixture of CaCO_3_/CaSO_4_. This investigation underscores the significant contribution of the carbon nanotubes’ (CNTs) intermediate layer in creating a durable superhydrophobic membrane, highlighting the potential of utilizing carbon nanotubes for tailored interface engineering to tackle fouling for salt mixtures. The innovative design of a superhydrophobic membrane has the potential to alleviate wetting issues resulting from low surface energy contaminants present in the feed of membrane distillation processes.

## 1. Introduction

In recent years, membrane distillation (MD) has gained significant promise in diverse applications such as water and wastewater treatment, desalination, and the recovery of volatile resources. MD is driven by a vapor pressure gradient existing between a hot feed and a cold permeate stream separated by a hydrophobic, microporous membrane while operating at temperatures below the boiling point [1,2]. MD also has the potential to be powered by low-grade heat sources such as solar, waste heat, or geothermal energy [3]. It has been well established that the microporous, hydrophobic membranes used in MD are less prone to fouling than RO which uses dense membranes, and that makes MD a technology of choice at higher fouling conditions [1,4,5,6,7,8].

Conventional microporous membranes, made from hydrophobic polymers like polyvinylidene fluoride (PVDF), polytetrafluoroethylene (PTFE), and polypropylene (PP), have certain limitations, and there is a need to develop low-fouling and wetting-resistant membranes [9,10,11,12,13,14]. The development of superhydrophobic and omniphobic membranes has the potential to enhance the performance of MD. The induction of superhydrophobicity establishes a vapor gap not only between water molecules but also on the membrane surface, potentially allowing for larger pore sizes before pore wetting ensues, thereby leading to increased flux. Additionally, the application of superhydrophobic modification holds the promise of reducing heat loss through conduction across the membrane [15,16,17,18,19]. 

The incorporation of hydrophobic nanoparticles, such as carbon nanotubes, reduced graphene oxide, boron nitride nanotubes, TiO_2_, ZnO, SiNP, etc., has been widely embraced in the preparation of hydrophobic membranes, offering potential avenues for further improvement based on the inherent superhydrophobicity exhibited by these particles [20,21,22]. Also, the development of micro-/nano-hierarchical structures on surfaces proves effective in enhancing surface roughness while surface energy can be reduced through the process of surface fluorination, achieved by silanization and the application of fluoroalkyl molecules [23,24]. Notably, the application of coatings such as fluorinated-decyl polyhedral oligomeric silsesquioxane (FD-POSS) and fluoroalkyl silane (FAS) onto polyester fabrics has resulted in a remarkable improvement in water, hexadecane, and tetradecane contact angles, reaching 171°, 155°, and 152°, respectively [25,26]. Other reports on membrane fluorination have explored the fabrication of electro-spun or phase-inversed substrates [27,28,29].

In recent developments, membranes based on carbon nanotubes (CNTs) referred to as carbon nanotube-immobilized membranes (CNIMs) have demonstrated remarkable separation performance [30,31,32,33]. This application has capitalized on the unique attributes of CNTs, such as their high aspect ratio, atomic-scale smoothness, chemical inertness, and sorption properties, that play a pivotal role in water molecule transport [34,35,36,37]. We have successfully engineered hydrophobic CNIMs and applied them across diverse separation processes, including membrane filtration [38,39,40], desalination [41,42,43], solvent recovery [44,45,46,47], pervaporation [48,49], and extraction [50,51]. In the arena of desalination, the immobilization of CNTs on the membrane surface dramatically alters the physicochemical interaction between water molecules and the membrane, enhancing flux in saline water treatment and potentially reducing scale formation on membrane surfaces. CNTs also act as a screen, resisting pore blocking by salt deposition, and facilitating the easy removal or washing off of salt crystals deposited on the screen-like CNTs [35,36,37]. In a recent study, we demonstrated that bilayer amphiphobic surfaces can be synthesized using CNTs for biomedical devices aimed at repelling blood plasma [52]. The hydrophobic nature of CNTs is inherent, and upon surface grafting with FAS, a thick and stable intermediate is formed, providing increased resistance to the permeation of organic compounds, thereby contributing to the wetting resistance of low surface tension liquids [44,53,54]. Superhydrophobic membranes incorporating CNT and FAS have been fabricated via electro-spinning [55], phase-inversion [55,56,57], and indirect fluorination of hydroxylated carbon nanotubes [27]. Recently, we have published bilayer membranes [44] that show strong anti-wetting properties for low surface tension liquids. Therefore, we hypothesize that a CNT intermediate layer that serves as a FAS grafting substrate could provide substantial benefits in robust resistance to rapid high fouling rates under simulated feed conditions with higher salt concentrations.

Here, the primary goal of this study was to develop and study the hierarchical membrane structure with a CNT intermediate layer that is coated with a low surface energy fluoroalkyl silane (FAS) layer to synthesize membranes (referred to as FAS-CNIM) to contribute superhydrophobicity and generate a higher flux, vapor permeability, fouling resistance, and thermal efficiency as applied to sweep gas membrane distillation (SGMD). Additionally, this research is aimed at investigating foulants such as CaSO_4_ and CaCO_3_. 

## 2. Experimental

### 2.1. Chemicals and Materials

The calcium sulfate (99%, pure), calcium carbonate (98%), ethanol, and acetone used in the experiments were purchased from Thermo Fisher Scientific (Hanover Park, IL, USA) and Sigma-Aldrich (St. Louis, MO, USA), while 1H,1H,2H,2H-Perfluorooctyltriethoxysilane, 97% (FAS), and Poly (vinylidene fluoride-co-hexafluoropropylene) (PVDF-HFP, Mw: 455,000 g/mol) were purchased from Sigma Aldrich (St. Louis, MO, USA). In all experiments, deionized water (Barnstead 5023, Dubuque, IA, USA) was used. The membrane used in these experiments was a flat composite PTFE membrane supported by polypropylene (0.45 µm pore size) which was purchased from Advantec, Dublin, CA, USA. Multi-walled carbon nanotubes (MWCNTs) were purchased from Cheap Tubes Inc., Brattleboro, VT, USA. The CNTs had an average diameter of ~30 nm and a length of 15 μm. 

### 2.2. Membrane Fabrication

This research involves the synthesis of two distinct membrane types: the CNIM and FAS-CNIM. The CNIM is composed of a PTFE membrane coated with carbon nanotubes (CNTs) using a method published before [44,52]. In the fabrication of the CNIM, approximately 3.8 mg of multi-walled CNTs were dispersed in 15 mL of acetone and subjected to three hours of sonication. Subsequently, PVDF-HFP (~0.043 g) was introduced to the dispersed solution, and the mixture underwent an additional hour of sonication. This process resulted in a homogeneous dispersion of PVDF/CNTs, preventing the entanglement of CNT nanoparticles. The prepared dispersion was carefully deposited in droplets onto the pristine PTFE membrane surface under vacuum conditions to ensure consistent distribution, adhering to a previously documented method. The excess PVDF-HFP was eliminated by flushing the membrane with acetone, and the membranes were left to dry overnight at room temperature for the evaporation of acetone. Adjustments to the quantities of CNTs and PVDF-HFP were made following several preliminary trials to achieve the increased water contact angle and comparable flux through the MD experiment. 

**Figure 1:**
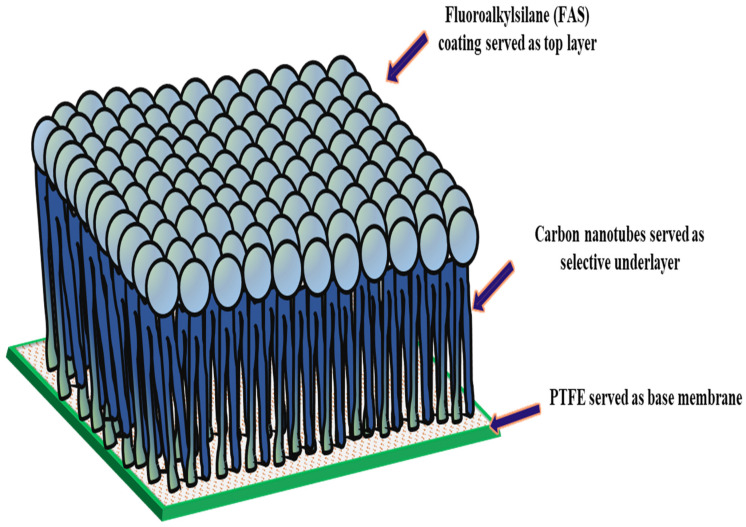
Structure of FAS-CNIM membrane.

In the structure of the FAS-CNIM, as depicted in Figure 1, the CNT coating functioned as the intermediary layer, while the FAS acted as the topmost layer. The application of the FAS layer involved dip coating the CNIM in a specific concentration of FAS within an ethanol solution. In this step, a 3% FAS solution was prepared by dissolving FAS in ethanol as reported in a previous study [44]. The CNT-coated CNIM underwent immersion in the FAS solution for up to 2 h, and this process was repeated through four continuous cycles, combined with air drying at room temperature. Exceeding this optimal FAS concentration and number of coating cycles did not result in significant improvements, indicating that the FAS coating on the membrane surface had reached saturation. Subsequently, the FAS-coated CNT membranes were subjected to heat treatment in an oven set at 160 °C for 1.5 h. Throughout the study, the unmodified membrane, designated as PTFE, served as the baseline across all sections.

### 2.3. Membrane Characterization

The membrane surfaces were characterized using scanning electron microscopy (SEM) (model JSM-7900F, JEOL USA Inc., Peabody, MA, USA) and Raman spectroscopy (Bruker Scientific DXR Raman microscope with a 532 nm wavelength laser and filter, Billerica, MA, USA). The membrane porosity was analyzed using the gravimetric method. Thermogravimetric analysis (TGA, Perkin Elmer 8000, Waltham, MA, USA) was used to analyze the thermal stability of the membranes. The liquid–membrane interactions were evaluated via contact angle measurements. The conduct of the feed and permeate was analyzed by a portable pH/conductivity/TDS meter (Apera Instruments, Columbus, OH, USA).

### 2.4. Experimental Setup

**Figure 2:**
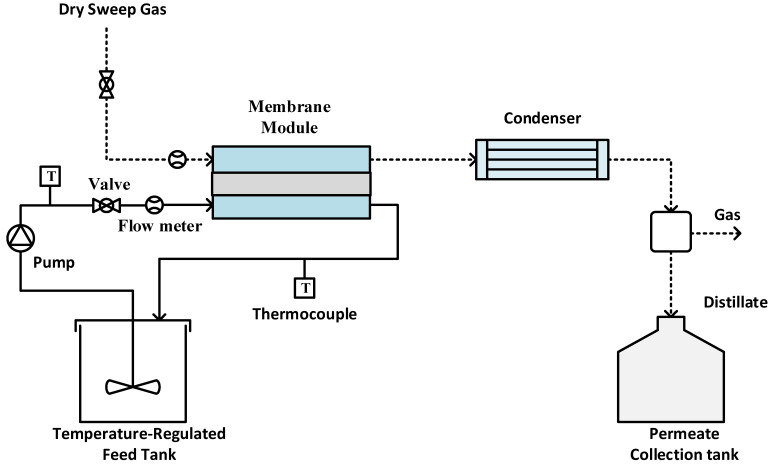
Experimental setup for SGMD.

Experiments were performed using an SGMD system, as illustrated in Figure 2. The sample membrane was securely positioned between two PTFE blocks, creating a flat sheet membrane module. The hot feed entered the module on the feed side, while dry gas swept through the permeate side to carry away the generated vapor [45,46,47]. The tubing was insulated to minimize heat loss during experiments. The module entrance and exit diameters are 0.5 cm; meanwhile, the groove space was 0.1 inch. The effective membrane surface area was 11.6 cm^2^. A peristaltic pump (Cole Parmer, model 77200-52, Vernon Hills, IL, USA) circulated the hot feed, with its temperature regulated by a constant temperature water bath. Temperature sensors monitored the feed and permeate temperatures. Dry compressed air at room temperature (22 °C) served as sweep gas to remove vapor from the permeate side. The flow rate on the permeate side was measured using a digital flow meter (Kelly Pneumatics, Inc., Newport Beach, CA, USA) and maintained at approximately 2 L/min for all experiments. In order to check the permeate quality, the electrical conductivity of the distillate was continuously monitored using a portable pH/conductivity/TDS meter (Apera Instruments, Columbus, OH, USA). The experiment was carried out using two different types of feed: a single-salt feed containing CaCO_3_ at a concentration of 2.8 g/L, and a mixed-salt feed comprising CaCO_3_ at 2.8 g/L and CaSO_4_ at 6.6 g/L.

## 3. Results and Discussion

**Figure 3:**
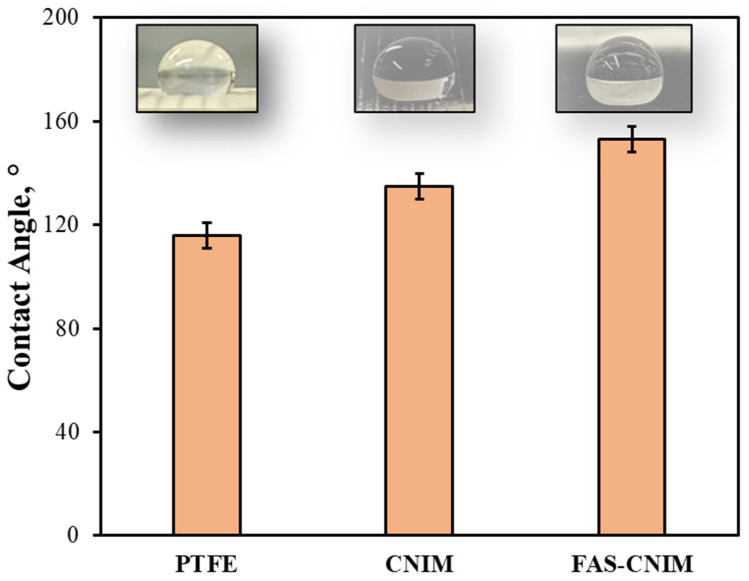
The contact angle of the PTFE, CNIM, and FAS-CNIM membranes.

**Figure 4:**
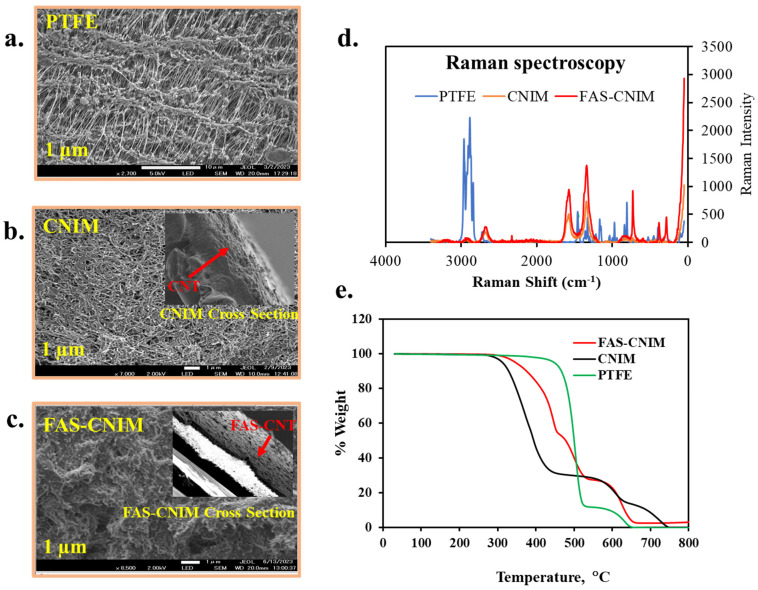
SEM images (**a**–**c**), Raman spectra (**d**), and thermogravimetric analysis of different membranes, namely PTFE, CNIM, and FAS-CNIM membranes (**e**).

The contact angles, SEM images, TGA, and Raman images of the membranes are shown in Figure 3 and Figure 4. The contact angle, defined as the angle formed between a liquid droplet and the membrane surface, provides insights into the interaction between the membrane and the liquid phase. A lower contact angle indicated favorable wetting, with the liquid spreading across the surface, while a higher angle suggested poor wetting, leading to bead formation. In Figure 3, the pristine PTFE membrane showed a contact angle of 118° and a CNIM angle of 135°, and the FAS-CNIM had a contact angle of 153°. The enhanced contact angle is attributed to the unique hydrophobic properties of the FAS layer.

Figure 4a–c show the SEM images for the PTFE, CNIM, and FAS-CNIM. The SEM image of the PTFE (Figure 4a) shows porous microstructure, Figure 4b shows well-dispersed CNTs, and Figure 4c shows the surface morphology of the FAS-CNIM where the FAS layer is clearly visible. The Raman spectra of the PTFE, CNIM, and FAS-CNIM are shown in Figure 4d. For the PTFE membrane, the Raman spectrum showed strong peaks related to carbon–fluorine bonds, emphasizing the absence of C-C or C-H vibrations due to its fully fluorinated structure. On the other hand, the CNTs exhibited distinctive Raman bands, including the G and the D band along with a characteristic D’ or 2D band to represent the graphitization degree of the CNT as the support material [58,59]. The Raman spectroscopy of the FAS-CNIM also shows a high intensity along with a slight shifting of the D, G, and 2D bands in Figure 4d which indicates the presence of the FAS layer on the top of the CNT layer in the membrane [60,61].

The incorporation of carbon nanotubes imparts enhanced thermal stability to the composite material, evident in a plateau or gradual weight loss region at higher temperatures in Figure 4e. This region signifies the resilience of carbon nanotubes against decomposition, contributing to the overall robustness of the CNT-immobilized membrane. This heightened thermal stability, a result of synergistic interactions between the polymer matrix and carbon nanotubes, augments the membrane’s performance. For modified membranes, there were four degradation zones in the curve specifying polypropylene, PTFE, FAS, and CNT. The polypropylene support in the pristine PTFE membrane degraded from 320 to 550 °C; meanwhile, PTFE degraded from 550 to 650 °C. However, the degradation trend was different for the FAS-CNIM. Between 320 °C and 530 °C, FAS and polypropylene degraded showing a mixed-temperature zone. PTFE started degrading at 530 °C and continued up to 680 °C. It was observed that the presence of CNTs provided better thermal stability to FAS-CNIM and degraded at a lower rate compared to other membranes [44,52].

The porosity of the modified membranes was also evaluated. The membrane porosity (ε) is defined as the volume of the pores divided by the total volume of the porous membrane. It can usually be calculated experimentally using the gravimetric method below [44,62,63]. The membrane samples were immersed in n-butyl alcohol for 48 h, and their size and thickness were measured using a precision micrometer. The observed membrane thicknesses were 130 ± 1 µm for unmodified PTFE, 150 ± 3 µm for the CNIM, and 155 ± 1 µm for the FAS-CNIM. The mass of the fully wetted sample and the dry sample was weighed using an electronic balance, and then the porosity was calculated according to the following:

(1) $$\varepsilon =\frac{w_{2}-w_{1}}{A\times D\times \rho }\times 100\%$$

where w_2_ and w_1_ were the weight of the wet and dry membranes, respectively, A was the area of the membrane sample (cm^2^), D was the average thickness of the membrane (cm), and *ρ* was the density of n-butyl alcohol (ρ = 0.81 g/mL). The result of the porosity measurement showed that the membrane modification did not alter the membrane porosity significantly; the PTFE had a porosity of 0.73; meanwhile, the CNIM had a porosity of 0.69, and the FAS-CNIM had a porosity of 0.68.

### 3.1. Desalination Performance of FAS-CNIM

The membrane performance was evaluated in terms of flux, rejection factor, and thermal analysis. Flux was measured at different concentrations and operating conditions and compared. The fouling behavior of the membranes was also evaluated for single salt and mixed salt. The water flux across the membrane is expressed as follows:

(2) $$J_{water}=\frac{W_{water}}{t\times A}$$

where *W_water_* is the amount of water vapor passed through the membrane at time *t*, and *A* is the effective membrane area exposed to sorption in the separation process. The amount of water permeating through the membrane was calculated from the weight of the collected permeate. The efficiency of separation for each membrane studied was represented by the rejection factor.

(3) $$\mathrm{Rejection}\;(\%),\;R=\frac{C_{feed,in}-C_{permeate,t}}{C_{feed,in}}\times 100\%$$

Here, *C_feed_* is the feed concentration of the salt, and *C_permeate_* is the concentration at the permeate side. 

**Figure 5:**
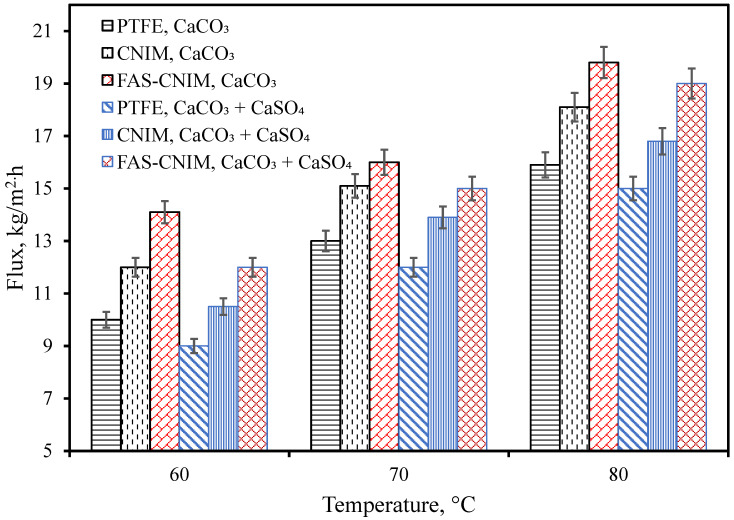
Membrane flux as a function of temperature for both a single salt (CaCO_3_, 2.8 g/L) and a mixture of salts (CaCO_3_ 2.8 g/L and CaSO_4_ 6.6 g/L) in the first hour of the SGMD study. The flow rate was maintained at 120 mL/min.

The flux for the PTFE, CNIM, and FAS-CNIM membranes are illustrated in Figure 5. In general, the water flux increased with temperature for all the membranes. The flux was evaluated using CaCO_3_ as well as CaCO_3_/CaSO_4_ mixtures. At all temperatures, CaCO_3_ containing water showed a higher flux compared to the mixed salts. Figure 5 shows that as the temperature changed from 60 °C to 80 °C, the flux increased from 14.1 kg/m^2^ h to 19.8 kg/m^2^ h in the FAS-CNIM for the CaCO_3_ solution. For the mixture of the CaCO_3_ and CaSO_4_ solution, the flux reached as high as 19 kg/m^2^ h at 80 °C for the FAS-CNIM. This increase in flux can be attributed to the robust anti-wetting property and preferential transport for the modified membranes. The FAS-CNIM exhibited a 26.7 and 12% increase in flux for the mixed-salt solution at 80 °C compared to PTFE and the CNIM, respectively. The FAS-CNIM also exhibited a 24.52 and 13.8% improvement in flux compared to PTFE and the CNIM for the CaCO_3_. This improved performance was attributed to the antifouling property of the FAS-CNIM and demonstrated the improved performance in dealing with mixed-salt solutions. Simultaneously, there was a decline in flux observed with mixed-salt solutions in contrast to single-salt solutions. This can be attributed to the elevated concentration of salt particles, which leads to a decreased vapor pressure, thereby limiting the amount of vapor able to pass through the membranes.

### 3.2. Fouling Characteristics of Membranes 

**Figure 6:**
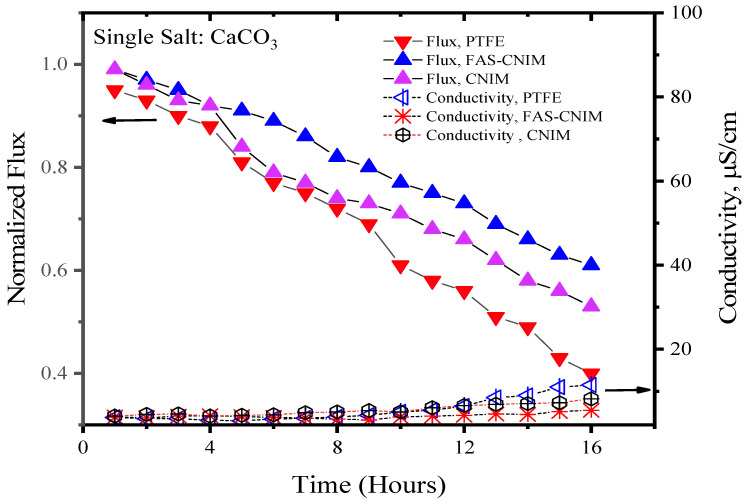
The fouling of membranes by CaCO_3_ at 80 °C for 16 h of the SGMD study. The flow rate was maintained at 120 mL/min.

**Figure 7:**
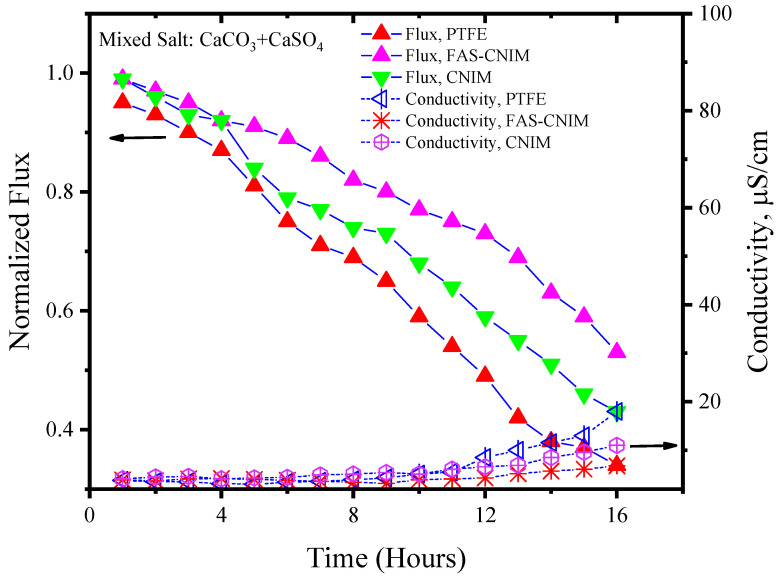
The fouling of membranes by CaCO_3_/CaSO_4_ at 80 °C for 16 h of the SGMD study. The flow rate was maintained at 120 mL/min.

Figure 6 and Figure 7 showcase the fouling behavior of salt solutions on both PTFE and modified membranes over a 16-h experimental period. Laboratory-grade milliQ water, with a conductivity of 6 µS/cm, was utilized to prepare the salt solutions. The initial conductivity of the CaCO_3_ solution was measured at 44 µS/cm, while the salt mixture consisting of CaCO_3_ and CaSO_4_ exhibited a conductivity of 2640 µS/cm. Notably, the mixed-salt solution typically displayed higher conductivity in the solution. Permeate quality was monitored using a conductivity meter at regular intervals. As fouling accumulated on the membrane surface over time, the flux demonstrated a consistent decreasing trend. Normalized flux, represented by the ratio of flux at any given time to its initial flux, exhibited a declining pattern for the PTFE, CNIM, and FAS-CNIM membranes, as evidenced by the downward trajectory of normalized flux lines in Figure 6 and Figure 7. However, discernible differences in final flux levels after 16 h were observed among the membranes. In the case of the CaCO_3_ solution, PTFE experienced an approximate 58% reduction in flux, followed by a 47% and 38% reduction in the CNIM and FAS-CNIM, respectively. Conversely, for the mixture of the CaCO_3_ and CaSO_4_ salts, the normalized flux declined by 69% for PTFE, 54% for the CNIM, and 43% for the FAS-CNIM. 

The permeate quality was monitored by measuring the conductivity of the permeate with respect to time. In the initial hours, the rejection performance was more than 99.5% and the conductivity was consistent with the milliQ water. As the fouling kept on depositing on the membrane surface, the rejection performance also decreased. Therefore, an increase in conductivity was seen in the permeate. After 16 h of SGMD with the CaCO_3_ solution, the permeate conductivity was 13 µS/cm for PTFE. The CNIM showed a conductivity of 10 µS/cm; meanwhile, FAS-CNIM exhibited a conductivity of 7 µS/cm. A similar trend was observed for mixed salts as well where the final conductivity was 19, 13, and 9 µS/cm, respectively, for the PTFE, CNIM, and FAS-CNIM at 80 °C. The long run of SGMD with excellent permeate quality also demonstrated the stability of the FAS-CNIM. 

**Figure 8:**
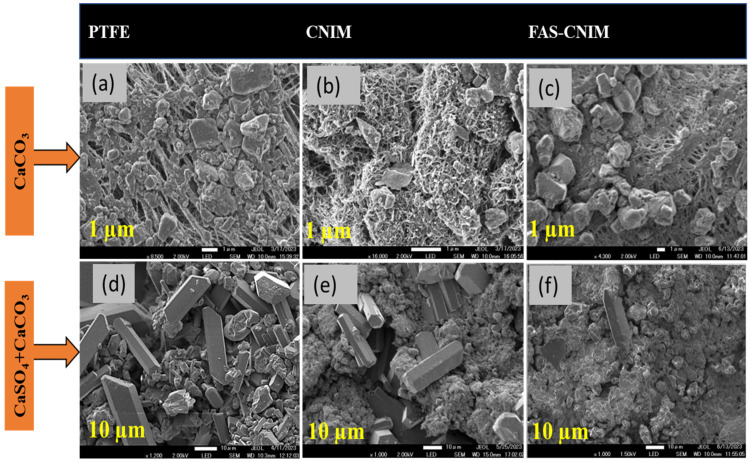
SEM images of the salt deposition after 16 h over the membrane surface. (**a**). CaCO_3_ salt deposition over the PTFE membrane surface. (**b**). CaCO_3_ salt deposition on the CNIM surface. (**c**). CaCO_3_ salt deposition on the FAS-CNIM surface. (**d**). CaCO_3_ and CaSO_4_ deposition on the PTFE membrane surface. (**e**). CaCO_3_ and CaSO_4_ deposition on the CNIM surface. (**f**). CaCO_3_ and CaSO_4_ deposition on the FAS-CNIM surface. The temperature for this fouling experiment was 80 °C with a flow rate of 120 mL/min. (Single salt: CaCO_3_ 2.8g/L, mixed salt: 2.8 g/L CaCO_3_ and 6.6 g/L CaSO_4_.).

The accumulation of salt on membrane surfaces, indicative of fouling, was assessed by quantifying the salt content over the experimental duration. Scanning electron microscopy (SEM) was employed to examine the morphological structure of the foulants, while gravimetric analysis was utilized to quantify the deposited foulant mass over time. SEM images of the membranes pre- and post-membrane distillation are depicted in Figure 8, where a, b, and c illustrate the fouling of the PTFE, CNIM, and FAS-CNIM membranes, respectively, in the presence of CaCO_3_. Figure 8d demonstrates more pronounced fouling when exposed to mixed salt, altering the morphology of the membranes compared to single-salt fouling. Conversely, Figure 8e,f depict the CNIMs and FAS-CNIMs fouled with the mixed salts, showing reduced fouling propensity compared to PTFE. Gravimetric measurements were employed to quantify the percentage of fouling deposition for all membranes.

The gravimetric analysis of the membranes for the fouling study is tabulated in Table 1. The relative flux reduction for the membranes was proportional to the amount of salt deposited. The PTFE membrane showed the highest amount of flux reduction for both single- and mixed-salt solutions. The FAS-CNIM exhibited 34.5% less reduction in relative flux compared to the PTFE membrane for the single-salt solution at a temperature of 80 °C. The salt deposit was 17.20 mg in this experiment. Meanwhile, for the mixed salt experiment, the FAS-CNIM showed a 43% drop in relative flux compared to a 69% drop in the PTFE membrane flux for mixed salts. The salt accumulation was also higher as shown in Table 1. This discrepancy in flux reduction originated from the intense fouling due to the presence of a salt mixture and salt crystal aggregation.

### 3.3. Energy Efficiency of MD Process

There are several parameters that can be used to measure thermal efficiency performance such as evaporation efficiency, specific energy consumption, and grained output ratio (GOR) [64,65]. Evaporation efficiency is defined by the amount of heat utilized for flux compared to the total energy passing through the membrane. Meanwhile, specific energy consumption shows how much energy is required per unit flux. On the other hand, the GOR is a measure of system efficiency comparing energy for flux generation to the input energy. The mathematical expression for each of the thermal efficiency parameters (TE) is given below [66], where Q_m_ is the heat transferred through the membrane by conduction and convection. J_p_ is the flux; A is the active membrane surface area, and H_v_ is the heat of vaporization for water.

(4) $$\mathrm{TE}\;(\%)=\frac{J_{p\;}\;A∆H_{v,w}}{Q_{m}}\times 100=\frac{Q_{vap}}{Q_{vap}+Q_{cond}}\times 100$$

Heat transferring through the membrane can be calculated using Equation (4) [67], where m_f_ is the feed flow rate, and C_p_ is the heat capacity of the water.

Q_m_ = m_f_ C_p_ (T_f, in_ − T_f, out_) (5)

Specific energy consumption [68]:

(6) $$\mathrm{SEC}\;(\mathrm{kWh}/m^{3})=[\frac{Q_{m}\;\rho }{J_{p}A}]/3600$$

Gained output ratio [69]:

(7) $$\mathrm{GOR}=\frac{J_{p\;}\;A∆H_{v,w}}{E_{in}}$$

E_in_ is the total energy provided in the system. Several assumptions were made to calculate the energy values. Feed heat capacity was assumed as water for simplification. Also, membrane conductivity was taken as the base PTFE membrane conductivity. This is because a small amount of nanocarbon is used for the modification of the membranes. The thermal efficiency for the PTFE, CNIM, and FAS-CNIM membranes are shown in Table 2. The evaporation efficiency was found to be highest for the FAS-CNIM; meanwhile, the specific energy consumption was the lowest. The GOR value for the mixed salt was 0.49 which is lesser than the single salt for the FAS-CNIM. This is attributed to an improved mass transfer contributed by the CNT layer.

## 4. Mechanism of Permeation in FAS-CNIM

**Figure 9:**
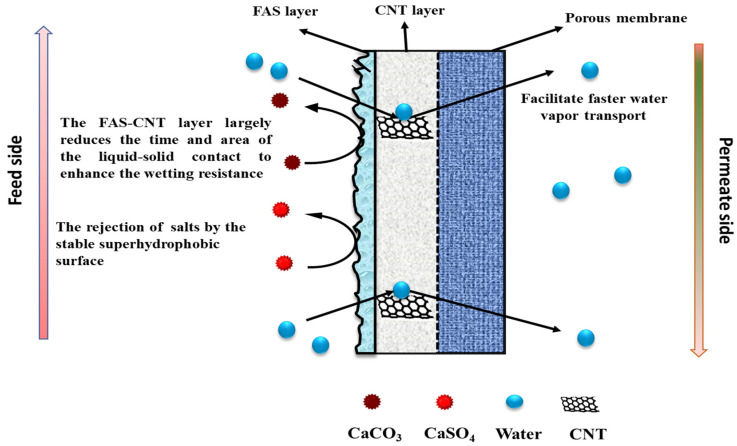
The mechanism of salt rejection and vapor transport through the membrane.

The underlying mechanism of the FAS-CNT layer in the desalination study is explained in Figure 9. The membrane has three different parts: a porous support layer at the bottom, a CNT layer in the middle, and a thin FAS layer at the top. The top FAS layer brings superhydrophobicity to the membrane surface along with the CNTs [70,71]. The fouling on the membrane surface depends on the initial concentration of feed, the nature of the feed, operating conditions, and the characteristics of the membrane surface. With a prolonged period of MD, the salt particles in the feed tend to deposit on the membrane surface [42]. Once the crystals are formed, the fouled layer tends to propagate quickly, especially at higher temperatures and salt concentrations. As a result, a decline in overall efficiency is observed. Because of the robust hydrophobicity of the FAS layer, the salt particles cannot propagate on the membrane surface. Also, the CNTs create an adsorptive pathway for the active transport of vapor molecules through the membrane surface. Therefore, there is no surface condensation of vapor molecules [42,72]. As a result of reduced fouling, a uniform temperature gradient is maintained along the membrane surface that ensures steady flux and high salt rejection [73]. 

## 5. Conclusions

In conclusion, this study has successfully demonstrated the efficacy of FAS-CNIMs in mitigating membrane fouling during desalination processes. The introduction of the FAS-CNT layer imparted superhydrophobicity to the membrane, achieving a contact angle as high as 153 ± 1°. This surface modification resulted in an 18% increase in membrane flux, coupled with a notable reduction in fouling even after prolonged exposure to high salt concentrations. A comparative analysis showed that mixed-salt solutions with CaCO_3_ and CaSO_4_ significantly increased membrane fouling, while the FAS-CNIM demonstrated enhanced performance under these conditions. The evaluation of thermal performance metrics such as specific energy consumption (SEC), evaporation efficiency, and gained output ratio further underscored the advantages of the FAS-modified membrane, with a 12% reduction in SEC observed compared to pristine PTFE membranes. Overall, these findings highlight the promising potential of FAS-modified membranes for effectively desalinating high salt concentrations, offering distinctive advantages in enhancing desalination processes.

## Figures and Tables

**Table 1:** The percentage flux reduction and fouling deposition on the membrane surface.

System	Relative Flux Reduction %, PTFE	Relative Flux Reduction %, CNIM	Relative Flux Reduction %, FAS-CNIM	Deposition of Salt PTFE (mg)	Deposition of Salt CNIM (mg)	Deposition of Salt FAS-CNIM (mg)
CaCO_3_	58 ± 2	47 ± 2.5	38 ± 1	17.20 ± 2	13.35 ± 1	10.1 ± 1
CaCO_3_ + CaSO_4_	69 ± 3	54 ± 2	43 ± 2	28.12 ± 1	21.2 ± 1	18.53 ± 1.5

**Table 2:** The thermal performance of the membranes during a desalination study for single- and mixed-salt mixtures.

Salt Mixture	Parameter	PTFE	CNIM	FAS-CNIM
CaCO_3_	TE (%)	66.2	74.5	82.7
	SEC (kwh/m^3^)	341	320	303
	GOR	0.41	0.46	0.51
CaCO_3_ + CaSO_4_	TE (%)	62	70.3	78.6
	SEC (kwh/m^3^)	354	330.5	311
	GOR	0.38	0.41	0.49

## Data Availability

Data is contained within the article or Supplementary Materials.

## Supplementary Materials

The following supporting information can be downloaded at: https://www.mdpi.com/article/10.3390/membranes14070152/s1, Figure: Real Experimental Set Up.
