# Supplementary material for: Fouling Reduction and Thermal Efficiency Enhancement in Membrane Distillation Using a Bilayer-Fluorinated Alkyl Silane–Carbon Nanotube Membrane
Source: Membranes (Basel). 2024 Jul 10;14(7):152. doi: 10.3390/membranes14070152 (PMC11279159; doi:10.3390/membranes14070152)
Supplement: Supplementary file 1 [file membranes-14-00152-s001.zip › membranes-3058255-supplementary.pdf]

Supporting Information

Fouling Reduction and Thermal Efficiency Enhancement in Membrane Distillation Using a Bilayer-Fluorinated Alkyl Silane–Carbon Nanotube Membrane

Sumona Paul, Mitun Chandra Bhoumick and Somenath Mitra *

Department of Chemistry and Environmental Science, New Jersey Institute of Technology,

Newark, NJ 07102, USA; sp2652@njit.edu (S.P.); mb777@njit.edu (M.C.B.)

* Correspondence: somenath.mitra@njit.edu;

Tel.: +1-973-596-5611; Fax: +1-973-596-3586

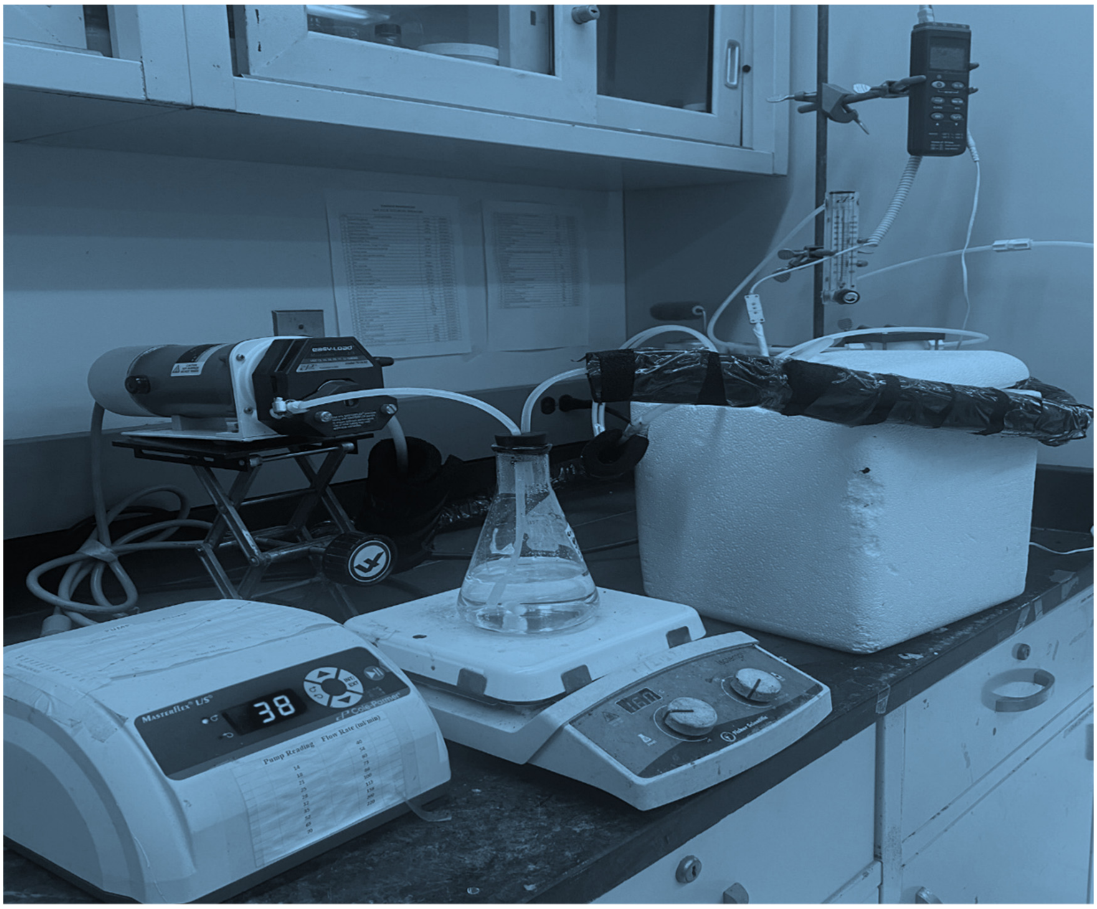

Figure: Real Experimental Set Up.
